# The effect of 17% EDTA and QMiX ultrasonic activation on smear layer removal and sealer penetration: *ex vivo* study

**DOI:** 10.1038/s41598-020-67303-z

**Published:** 2020-06-25

**Authors:** Felipe de Souza Matos, Fabrício Rutz da Silva, Luiz Renato Paranhos, Camilla Christian Gomes Moura, Eduardo Bresciani, Marcia Carneiro Valera

**Affiliations:** 1 Postgraduate Program in Dentistry, School of Dentistry, Federal University of Uberlândia (UFU), Uberlândia, MG Brazil; 2 Department of Restorative Dentistry, School of Dentistry, State University of Ponta Grossa (UEPG), Ponta Grossa, PR Brazil; 3 Department of Preventive and Social Dentistry, School of Dentistry, Federal University of Uberlândia (UFU), Uberlândia, MG Brazil; 4 Department of Endodontics, School of Dentistry, Federal University of Uberlândia (UFU), Uberlândia, MG Brazil; 5 Department of Restorative Dentistry, Institute of Science and Technology, São Paulo State University (Unesp), São José dos Campos, SP Brazil

**Keywords:** Endodontics, Root canal treatment

## Abstract

This study aimed to compare the effect of conventional irrigation (CI) and passive ultrasonic irrigation (PUI) with 17% EDTA and QMiX on the maximum depth and percentage of sealer penetration into the dentinal tubules by confocal laser scanning microscopy (CLSM) and to describe the cleaning of root canal walls by scanning electron microscopy (SEM). Eighty single-rooted human mandibular premolars were instrumented and randomly assigned to four groups (n = 20): EDTA + CI, QMiX + CI, EDTA + PUI, and QMiX + PUI. Ten samples from each group were examined by SEM (2,000×) and the remaining 40 roots were filled with a single gutta-percha cone and AH Plus sealer mixed with 0.1% rhodamine B for analysis by CLSM (10×). Images were assessed at distances of 2 mm (apical), 5 mm (middle), and 8 mm (coronal) from the apex with the Leica Application Suite V4.10 software. The EDTA + PUI and QMiX + PUI protocols presented higher rates of debris/smear layer removal in the apical and middle thirds. The PUI was superior to CI in the maximum depth of sealer penetration at the middle third. The QMiX + PUI group had a higher percentage of sealer penetration at the apical third. The PUI and QMiX protocol improved debris/smear layer removal and tubular dentin sealer penetration.

## Introduction

The success of endodontic therapy depends on the chemomechanical disinfection and appropriate antimicrobial sealing of the root canal system (RCS)^1^. The biomechanical preparation using mechanical instrumentation and antimicrobial solutions aims to shape the root canal and either eliminate or reduce toxic and necrotic contents, including pulp remains and pathogens^2^. However, as a result of instrumentation, a 1- to 2-µm thick smear layer primarily composed of inorganic dentin is formed in the root canal walls and it should be removed during the final irrigation with chelating agents because it blocks the dentinal tubules, harboring necrotic debris and bacteria and their by-products^3^. It also limits the penetration of disinfectants and sealers into the dentinal tubules^4^.

Sodium hypochlorite (NaOCl) is the main irrigating solution used in the endodontic treatment due to its antimicrobial action and solvent capacity on organic tissues, but it does not affect the inorganic content. To remove debris and smear layer and improve the permeability of the RCS, final irrigation with ethylenediaminetetraacetic acid (EDTA) is recommended as a prerequisite for a satisfactory sealing of the dentinal canaliculi^5^. More recently, the final irrigant QMiX has gained special attention for presenting a chelating effect similar or superior to EDTA, besides antimicrobial activity^6–9^. The QMiX promotes a superior sealer penetration to that achieved by other chelating solutions such as BioPure MTAD^10^.

Although the antimicrobial and chelating actions of endodontic irrigants play a critical role in disinfecting and cleaning the RCS, conventional needle irrigation may not allow these substances to work deep into the dentinal tubules. Thus, different devices and irrigant activation techniques have been developed and recommended to improve the efficiency and distribution of solutions^11–13^. Passive ultrasonic irrigation (PUI) activates the irrigant solution by acoustic microstreaming transmitted from an oscillating file or smooth wire at an ultrasonic frequency of 30 kHz. It also improves the cleaning and disinfection of the RCS when compared to conventional needle irrigation^11,14^.

Several studies have shown that the proper filling of the RCS depends on the chelating capacity of chemicals used mainly during the final cleaning and on the concomitant use of some activation systems^15–17^. Although some studies report that QMiX and 17% EDTA exhibit similar root canal cleaning ability^6^, the effect of different activation techniques of these substances on smear layer removal and sealer penetration has not yet been well explained^11,17,18^. The penetration of sealer into the dentinal tubules is clinically important because adequate sealing may control infections and prevent recontamination^19^.

The effect of the combined use of QMiX and PUI on both the cleaning of root canal walls and sealer penetration into the dentinal tubules has not been studied to date. Therefore, the present study was conducted to compare the effect of conventional irrigation (CI) and passive ultrasonic irrigation (PUI) with 17% EDTA and QMiX on the maximum depth and percentage of sealer penetration into the dentinal tubules by confocal laser scanning microscopy (CLSM). It also aims to describe the cleaning of root canal walls by scanning electron microscopy (SEM) at different root canal levels (cervical, middle, and apical thirds). The authors tested the following hypotheses: (1) There is no difference in the rate of debris/smear layer removal between conventional or passive ultrasonic irrigations with 17% EDTA and QMiX; (2) There is no difference in the rate of tubular dentin sealer penetration between conventional or passive ultrasonic irrigations with 17% EDTA and QMiX.

## Material and Methods

### Sample size

This study was approved by the Research Ethics Committee of the Institute of Science and Technology – Unesp (Certificate of Presentation for Ethical Consideration: 79730317.2.0000.0077) and the methods were carried out in accordance with the Declaration of Helsinki (2008). Sample size calculation was performed based on previous data^10^ with the G*Power software (version 3.1), using the following parameters: two-tailed 5% significance level (α = 0.05), 95% confidence interval, 90% statistical power (β = 0.10), 1:1 ratio of specimen allocation in the experimental groups, and medium estimated effect size (d = 0.60), which indicated the need to include a minimum of 20 specimens in each group. Thus, a final study sample of 80 human mandibular premolars freshly extracted for orthodontic or periodontal reasons were used. The teeth were collected from the Department of Surgery, Periodontics and Radiology of the Institute of Science and Technology – Unesp, and all patients have obtained the informed consent.

### Sample selection

The teeth were immersed in 0.1% thymol solution during 48 hours for disinfection and stored in distilled water at 4 °C until use. All the teeth were evaluated radiographically to confirm the presence of a single root canal, mature apex, and absence of any resorption or endodontic treatment. The study included only teeth with radicular canal widths of 3–4 mm buccolingually and 1–2 mm mesiodistally at the cementoenamel junction level, anatomic diameter of the #15 or #20 Kerr file (Dentsply Maillefer, Ballaigues, Switzerland) at 1 mm from the apex, and straight or curved canal up to 10°^20^. The teeth selected were cross-sectioned in their long axes below the cementoenamel junction for removing the crown and standardizing the total root length at 12.0 ± 0.5 mm, using a diamond disc (Horico Dental Hopf, Ringleb & Co GmbH & Cie, Berlin, Germany).

### Root canal preparation

The full length of the root canals was instrumented up to a #30 Kerr file (Dentsply Maillefer, Ballaigues, Switzerland) using 3 mL of saline solution after each instrumentation to standardize the apical diameter. The canals were filled with 17% EDTA (Inodon, Porto Alegre, RS, Brazil) for three minutes and irrigated with 10 mL of saline solution. Next, the apical foramen was sealed with light-cured composite resin (Z-100, 3 M, Saint Paul, USA) to create an apical seal. The biomechanical instrumentation was performed with the R40 WaveOne single-file reciprocating system (Dentsply Maillefer, Ballaigues, Switzerland) according to the crown-down technique (i.e., coronal, medium, and apical) associated with irrigation of 5 mL of 2.5% sodium hypochlorite (NaOCl) (Fórmula e Ação, São Paulo, Brazil) for each third, totaling 15 mL for each sample. The apical working length (WL) was established at 1 mm short of the anatomical apex and irrigation was performed using a 5-mL disposable syringe (Ultradent, South Jordan, USA) and a 30-gauge NaviTip needle (Ultradent, South Jordan, USA) inserted into the canal 2 mm short of the WL. Foraminal patency was maintained with a #15 Kerr file. The root canals were rinsed with 10 mL of saline solution to neutralize NaOCl.

### Final irrigation

After instrumentation, the samples were randomly assigned to four groups (n = 20 each) according to the final irrigation protocol used: conventional irrigation (CI) or passive ultrasonic irrigation (PUI) with 17% EDTA and QMiX, which are described below.

EDTA + CI. The samples were flooded with 17% EDTA solution (Inodon, Porto Alegre, RS, Brazil) for two minutes without any type of agitation.

QMiX + CI. The samples were flooded with QMiX (Dentsply Tulsa Dental Specialties, Johnson City, TN, USA) for two minutes without any type of agitation.

EDTA + PUI. The samples were flooded with 17% EDTA solution (Inodon, Porto Alegre, RS, Brazil) for two minutes and ultrasonically agitated in the last 60 seconds.

QMiX + PUI. The samples were flooded with QMiX (Dentsply Tulsa Dental Specialties, Johnson City, TN, USA) for two minutes and ultrasonically agitated in the last 60 seconds.

All root canals were irrigated with 3 mL of the respective final irrigating solution using a 5-mL disposable syringe and a 30-gauge NaviTip needle placed into the canal 2 mm short of the WL. The ultrasonic activation was performed at a minimum power setting (10%), using an Irrisonic tip (Helse, Santa Rosa do Viterbo, SP, Brazil) inserted into the canal 1 mm short of the WL. After removing the excess of the final irrigant, the canals were rinsed with 10 mL of saline solution and dried with paper points.

### Scanning electron microscopy evaluation

To describe the effect of the final irrigation protocol on the removal of debris and smear layer from the root canal walls, 10 samples from each group were prepared for scanning electron microscopy (SEM). Two longitudinal grooves were made along the external root surface in the mesiodistal direction using a diamond disc, and the samples were split in half with a chisel to expose the root canal. The root halves were dehydrated in ascending ethanol and dried in an incubator at 37 °C for 24 hours. Subsequently, they were mounted on stubs and coated with 20-nm thick gold-palladium for the SEM evaluation. One representative image of the apical (2–3 mm), middle (5–4 mm), and coronal (8–9 mm) thirds from each sample were obtained at a magnification of 2,000×, totaling 120 images. The efficacy of debris and smear layer removal was evaluated blindly by two investigators using a four-level scoring system: 0 = no debris/smear layer with all tubules open; 1 = minimum quantity of debris/smear layer with over 50% of the tubules open; 2 = moderate quantity of debris/smear layer with less than 50% of the tubules open; 3 = heavy debris/smear layer with almost all dentin tubules obstructed^21^. The investigators were previously calibrated for the scoring system, which they applied to a sample of 20% of the SEM images randomly selected from two specimens of each group (n = 24) to determine the inter-examiner agreement. After achieving a proper level of agreement (Kappa≥0.81), the investigators scored the images independently. The lowest score was chosen when there were conflicting results among the investigators. The scores were statistically evaluated using the Kruskal-Wallis test followed by Dunn’s test, at 5% significance level.

### Root canal filling

The remaining 40 roots were filled with AH Plus sealer (Dentsply; DeTrey, Konstanz, Germany) and a single R40 WaveOne gutta-percha cone (Dentsply Maillefer, Ballaigues, Switzerland) according to their manufacturers’ instructions. The sealer was mixed with 0.1% fluorescent rhodamine B isothiocyanate dye (Sigma-Aldrich, St Louis, MO, USA) to allow the analysis under confocal microscope and it was placed into the canal using a #25.02 Lentulo spiral (Dentsply Maillefer, Ballaigues, Switzerland) attached to a handpiece at 20,000 rpm, inserted into the canal 1 mm short of the WL for five seconds. The excess of gutta-percha was removed with a heated Schilder™ plugger (Dentsply Maillefer, Ballaigues, Switzerland) and vertical compaction was performed at the orifice level. The coronal access was sealed with light-cured composite resin and the samples were stored in an incubator at 37 °C and 100% humidity for seven days to allow the sealer to set completely^10^.

### Confocal laser scanning microscope evaluation

**Figure 1:**
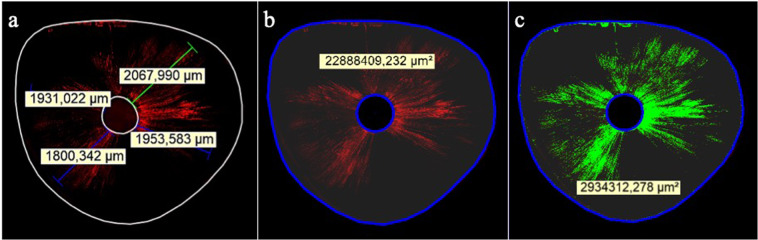
Maximum depth and percentage of sealer penetration: (**a**) Four measurements of the penetration depth were recorded in the slice and maximum penetration was considered (green line); (**b**) The total area of the canal wall was recorded; and (**c**) Areas of sealer penetration were measured (green areas).

Each sample was sectioned perpendicular to its long axis in three 1-mm thick slices using a diamond blade in IsoMet (Buehler, Illinois, USA) at distances of 2 mm (apical), 5 mm (middle), and 8 mm (coronal) from the apex. The coronal surfaces of the slices were polished using silicon carbide abrasive papers to eliminate dentin debris produced during the cutting procedure. Then, they were mounted onto glass slides and examined under a Leica TCS SP5 confocal laser scanning microscope (Leica Microsystems, Mannheim, Germany) at a magnification of 10× and wavelength of 540–590 nm. The Leica Application Suite V4.10 software (Leica Microsystems Ltd., Heerbrugg, Switzerland) was used to measure the maximum depth and percentage of sealer penetration into the dentinal tubules with a calibrated measuring tool (Fig. 1). The maximum depth of sealer penetration was measured from the root canal wall to the point of deepest penetration in the dentinal tubules (Fig. 1a). Areas along the root canal walls in which the sealer had penetrated were measured and divided by the total area along the canal walls to determine the percentage of sealer penetration (Fig. 1b,c). The means of percentage and maximum depth of sealer penetration for each root canal level were evaluated using ANOVA followed by Tukey’s test, at 5% significance level.

## Results

### Scanning electron microscopy evaluation

**Figure 2:**
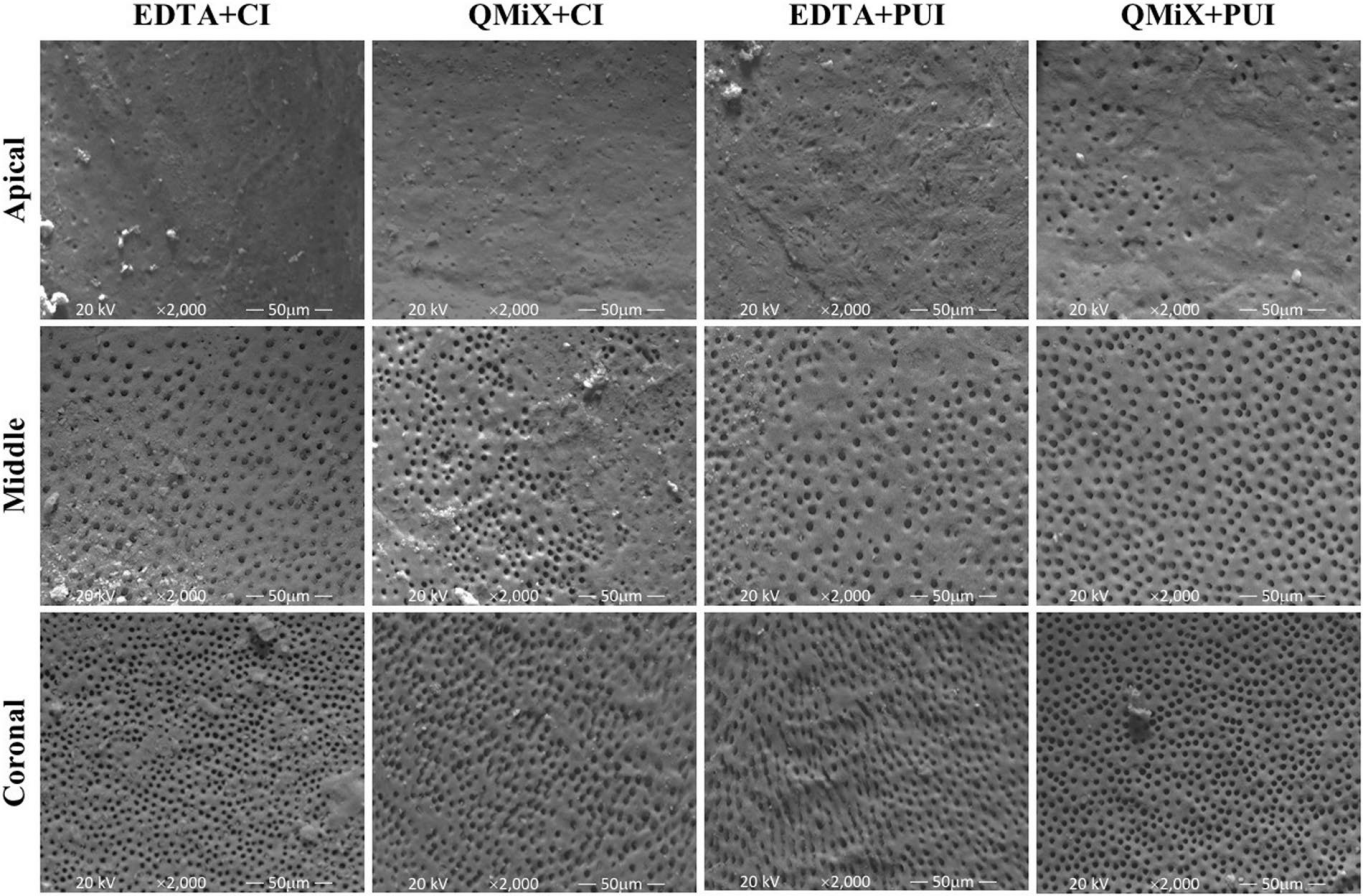
Representative SEM images of debris and smear layer removal from the root canal walls in each group and root third (original magnification, ×2,000).

The Kappa test showed an almost perfect level of agreement between the investigators (Kappa=0.923). Table 1 summarizes the results of the evaluation of debris and smear layer removal. Lower scores for debris/smear layer removal were achieved by the EDTA + PUI and QMiX + PUI groups in the apical and middle thirds when compared with the EDTA + CI and QMiX + CI groups (p < 0.05). There were no statistically significant differences among the protocols in the coronal third (p > 0.05). In the EDTA + CI and QMiX + CI groups, the greatest debris/smear layer removal was observed in the coronal third, followed by the middle and apical thirds, respectively (p < 0.001) (Table 1). In the EDTA + PUI and QMiX + PUI groups, a higher debris/smear layer removal was obtained in the coronal and middle thirds than in the apical third (p < 0.001) (Table 1). Figure 2 shows representative SEM images of debris and smear layer removal from the root canal walls in each root third after the final irrigation protocols.

**Table 1:** Comparison among groups considering the scores of debris/smear layer removal (median and IQR) in each root canal third.

Groups	Apical third	Middle third	Coronal third	*p* value
EDTA + CI	3 (3; 3) ^aA^	2 (1; 2) ^aB^	1 (0; 1) ^aC^	< 0.001
QMiX + CI	3 (3; 3) ^aA^	2 (1; 2) ^aB^	1 (0; 1) ^aC^	< 0.001
EDTA + PUI	2 (2; 3) ^bA^	1 (1; 1) ^bB^	1 (0; 1) ^aB^	< 0.001
QMiX + PUI	2 (2; 3) ^bA^	1 (0; 1) ^bB^	1 (0; 1) ^aB^	< 0.001
*p* value	< 0.05	< 0.05	> 0.05	

Dunn’s test (multiple comparisons): different lowercase letters represent statistically significant difference among groups (p < 0.05) and different capital letters represent statistically significant difference among thirds (p < 0.05). IQR = interquartile range.

### Confocal laser scanning microscope evaluation

**Figure 3:**
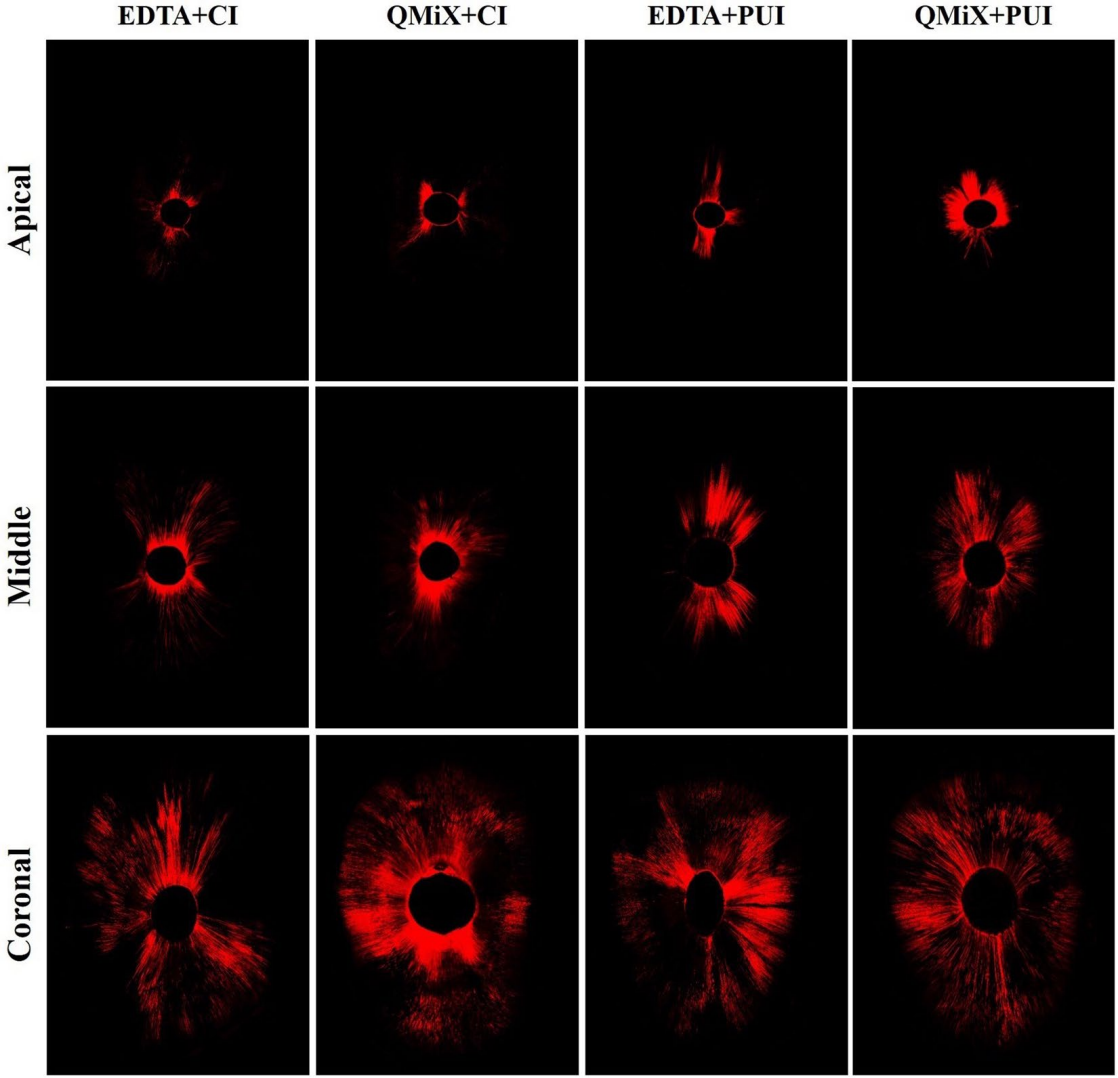
Representative CLSM images of sealer penetration around the root canal walls in each group and root third (original magnification, ×10).

Tables 2 and 3 present the means and standard deviations of maximum depth and percentage of sealer penetration at each root canal third, respectively. There was no statistically significant difference in the maximum depth of sealer penetration among the final irrigation protocols in the apical and coronal thirds (p > 0.05). There was a statistically significant difference in the middle third between PUI and CI for both final irrigating solutions, with better results for the EDTA + PUI and QMiX + PUI groups (p < 0.001). In all groups, the greatest depth of sealer penetration was observed in the coronal third, followed by the middle and apical thirds, respectively (p < 0.001) (Table 2). Figure 3 shows representative patterns of sealer penetration around the root canal walls in each group and root third.

**Table 2:** Comparison among groups considering the maximum depth of sealer penetration in µm (mean ± SD) in each root canal third.

Groups	Apical third	Middle third	Coronal third	*p* value
EDTA + CI	684 ± 175 ª^A^	1391 ± 164 ª^B^	1927 ± 303 ª^C^	< 0.001
QMiX + CI	677 ± 211 ª^A^	1392 ± 218 ª^B^	1951 ± 191 ª^C^	< 0.001
EDTA + PUI	690 ± 174 ^aA^	1743 ± 177 ^bB^	2162 ± 191 ª^C^	< 0.001
QMiX + PUI	847 ± 148 ª^A^	1702 ± 152 ^bB^	2113 ± 167 ª^C^	< 0.001
*p* value	> 0.05	< 0.001	> 0.05	

Tukey’s test (multiple comparisons): different lowercase letters represent statistically significant difference among groups (p < 0.05) and different capital letters represent statistically significant difference among thirds (p < 0.05). SD = standard deviation.

**Table 3:** Comparison among groups considering the percentage (%) of sealer penetration (mean ± SD) in each root canal third.

Groups	Apical third	Middle third	Coronal third	*p* value
EDTA + CI	11.14 ± 1.22 ^aA^	21.48 ± 2.97 ^aB^	27.30 ± 7.21 ^aC^	< 0.001
QMiX + CI	11.07 ± 1.01 ^aA^	24.10 ± 3.42 ^aB^	35.65 ± 7.08 ^bC^	< 0.001
EDTA + PUI	14.31 ± 3.53 ^bA^	24.70 ± 2.77 ^aB^	33.58 ± 6.85 ^abC^	< 0.001
QMiX + PUI	20.85 ± 2.38 ^cA^	24.60 ± 2.91 ^aA^	35.03 ± 5.26 ^abB^	< 0.001
*p* value	< 0.001	> 0.05	< 0.05	

Tukey’s test (multiple comparisons): different lowercase letters represent statistically significant difference among groups (p < 0.05) and different capital letters represent statistically significant difference among thirds (p < 0.05). SD = standard deviation.

Regarding the percentage of sealer penetration, there was no statistically significant difference among the final irrigation protocols only in the middle third (p > 0.05). At the apical third, QMiX + PUI presented a significantly higher percentage of sealer penetration than the other groups (p < 0.001), followed by EDTA + PUI and conventional irrigation with EDTA or QMiX, with no statistical difference between EDTA + CI and QMiX + CI (p > 0.05). The groups showed similar percentages of sealer penetration in the coronal third, but QMiX + CI performed better than EDTA + CI (p < 0.05). As in the previous analysis, the coronal third presented the highest percentage values of sealer penetration around the root canal walls, regardless of the final irrigation protocol, followed by the middle and apical thirds, respectively (p < 0.001). However, in the QMiX + PUI group, the apical and middle thirds presented statistically similar percentages of sealer penetration (Table 3 and Fig. 3).

## Discussion

The quality of root canal cleaning and filling is a significant predictor of successful endodontic treatment^1^. According to Chugal *et al*., a poor root filling may increase about twice the rate of treatment failure of teeth without periapical lesion, and this rate is 20% higher for teeth with apical periodontitis^1^. The presence of smear layer contributes not only to the ineffective obturation^16^ but also to the adhesion and colonization of bacteria and endotoxins in the dentin matrix^22^. The sealer penetration into the dentinal tubules is desirable because it might bury residual microorganisms and their toxins, keeping them away from nutrient sources and preventing reinfection^23^. Thus, considering the influence of the quality of final cleaning and obturation on the endodontic outcome, this study evaluated the effect of conventional irrigation (CI) or passive ultrasonic irrigation (PUI) with 17% EDTA and QMiX on debris/smear layer removal and tubular dentin sealer penetration in *ex vivo* human root canals. Hypothesis (1) and (2) were rejected, as the final irrigation protocols tested differ in their effects on debris/smear layer removal and tubular dentin sealer penetration.

The results of this study showed that PUI with both 17% EDTA and QMiX final irrigants improved the removal of debris/smear layer in the middle and apical thirds of the root canal when compared to CI. These findings are consistent with other studies that observed a greater smear layer removal when supplementing the final irrigation with PUI^11,18,24–26^. The smear layer is an amorphous structure containing mostly inorganic dentin debris and organic substances such as fragments of the odontoblastic process, microorganisms, and necrotic pulp tissue^4^. Ultrasonic activation potentially improved the debris/smear layer removal by causing shear stress in the inorganic particles of the smear layer by acoustic streaming, facilitating its removal^14^. However, the irrigant delivered by conventional needle only penetrates from 0 to 1.1 mm deeper than the tip of the needle, and gas particles are produced and trapped in the apical portion, creating a vapor lock and hindering the efficacy of irrigant debridement^12^. On the other hand, PUI allows the elimination of vapor lock effect, improving the efficiency of the irrigating solution^25^. The safety of CI has also been questioned because the positive pressure used to deliver the solution into the canal may extrude it to the periapex, causing tissue damage and postoperative pain^27^.

The mechanism of action of chelating solutions is based on their ability to react with calcium ions in dentin and to form soluble calcium chelates^4^. The 2-minute working time adopted in this study for both irrigants was based on a previous study^7^ that observed positive antimicrobial and detoxifying effects of QMiX also at 2 minutes. The QMiX contains EDTA, CHX, and cetrimide, a detergent that decreases surface tension and increases wettability and penetrability^6^. However, similar to the results from earlier studies, the QMiX was as effective as 17% EDTA in debris/smear layer removal^6,10,18^. In turn, Nogo-Zivanovi *et al*. and Vemuri *et al*. showed that QMiX removes the smear layer in the apical third more effectively than 17% EDTA^8,28^. Souza *et al*. revealed that ultrasonic activation of QMiX was significantly more effective in removing the smear layer in the cervical third than ultrasonic activation of 17% EDTA^26^. Hence, there is still no consensus as to which chelating solution is the most efficient. Differences in experimental design may help to explain the disparate results obtained in the studies mentioned, especially regarding the anatomical particularities of the specimens used, the volume of solution, and time spent for irrigation. Although the protocol recommended is the use of NaOCl after smear layer removal with EDTA to kill the remaining bacteria, this regimen was not used in this study because NaOCl after EDTA causes more dentin erosion^29^; also the use of NaOCl might mask the effect of the solutions tested. Additionally, because the composition of QMiX contains an antimicrobial agent, it exempts the final rinse with NaOCl^6^.

The effect of final irrigation with QMiX on tubular dentin sealer penetration was compared with 17% EDTA in previous studies^10,30,31^. However, to our knowledge, this is the first study to report the effects of PUI with QMiX on tubular dentin sealer penetration. The single cone technique was used in this experiment because of its wide use in endodontics and because the filling technique does not interfere with the penetration capacity of sealers^32^. According to our results, PUI improved the maximum depth of sealer penetration in the middle third of the canal when compared to CI, regardless of the chelating solution, which agrees with previous studies using 17% EDTA^15,16^. The best results for the percentage of sealer penetration were obtained for QMiX + PUI only in the apical third. This may be a direct effect of a better debris/smear layer removal found in the groups treated with PUI in both the middle and apical thirds, confirming the assumption that sealer penetration into the dentinal tubules may be an indicator for the cleaning quality of the canal^10^. Representative SEM images (Fig. 2) showed greater debris/smear layer removal in the groups with the highest percentage of sealer penetration (QMiX + PUI, apical third), with almost all tubules opened and a minimum amount of debris/smear layer attached to the canal walls. The ability of QMiX to promote a higher percentage of sealer penetration may be related to its chemical design containing surfactant (detergent), which increases irrigant flow in the root canal and its contact with the smear layer, improving dentin permeability^8^. In contrast, 17% EDTA has no detergent in its composition and presents high surface tension and low permeability, which limits its chelating effect^33^. Interestingly, other studies showed that 17% EDTA and QMiX promoted similar sealer penetration^10,30,31^.

Overall, the efficacy of the final irrigation protocols for debris/smear layer removal and sealer penetration decreased from the coronal to the apical thirds. This may have been due to two factors. First, the number and diameter of dentinal tubules decrease toward the apical third^34^ and, second, the apical third presents more sclerotic dentin and greater difficulty for irrigant delivery and smear layer removal, which has a direct effect on sealer penetration^12,23^. This result agrees with previous studies that showed that irrigating solutions are less effective in the apical third^8,15,18,30,31^. However, in contrast to CI, no significant difference was observed between the middle and coronal thirds in the debris/smear layer removal for the EDTA + PUI and QMiX + PUI protocols and between the middle and apical thirds in sealer penetration for the QMiX + PUI protocol. This result indicates that both ultrasonic activation and QMiX improved the cleaning and tubular dentin sealer penetration in the middle and apical thirds, respectively. This may be due to the synergistic effect of the ultrasound and the presence of detergent in QMiX that potentially increases its effectiveness by favoring its action on a larger surface area in the root canal and deeper into the dentinal tubules^8^.

The results of this *ex vivo* study show a close relationship between the final cleaning level of the root canal and tubular dentin sealer penetration. Particularly in the apical third, the QMiX + PUI final irrigating protocol provided an improved debris/smear layer removal of the canal walls and a higher percentage of sealer penetration into the dentinal tubules. This finding is important because the apical third is considered the critical region of the root canal for presenting a greater amount of ramifications of the main root canal. These ramifications are inaccessible to the conventional chemomechanical preparation, which allows harboring remaining bacteria and their by-products and leads to the failure of the endodontic therapy^35,36^. Thus, the present study reveals that final irrigation protocols using PUI and a chelating solution such as QMiX may improve the quality of the cleaning and obturation of the root canal system and it should be considered in the daily clinical practice. However, the level of evidence of the SEM method is limited because only a very small part of the root canal can be evaluated and used to represent the sample^14^. The method of sectioning root dentin samples for analysis in confocal microscopy is already well established as an effective technique that allows determining the presence and extent of penetration of the root canal sealer into the dentinal tubules^15,23^. The major limitation of the present study relates to the laboratory character of the experiment, which restricts extrapolating the results for the clinical practice. Clinical studies are required to validate our results and to evaluate the effect of the final irrigation protocols tested on the clinical success of endodontic treatment.

## Conclusion

Passive ultrasonic irrigation improved debris/smear layer removal and sealer penetration into the dentinal tubules in the apical and middle thirds. When all factors were considered (final irrigating solution, irrigation technique, root canal thirds, debris/smear layer removal, and sealer penetration), the passive ultrasonic irrigation with QMiX showed the best results.

## Data Availability

The datasets generated during and/or analyzed during the current study are available from the corresponding author on reasonable request.
